# Design and biological characterization of novel cell-penetrating peptides preferentially targeting cell nuclei and subnuclear regions

**DOI:** 10.3762/bjoc.14.116

**Published:** 2018-06-07

**Authors:** Anja Gronewold, Mareike Horn, Ines Neundorf

**Affiliations:** 1 Department of Chemistry, Biochemistry, University of Cologne, Zuelpicher Str. 47a, 50674 Cologne, Germany

**Keywords:** anticancer drugs, cell nuclei, cell-penetrating peptides, nucleoli, subcellular targeting

## Abstract

Within this study, we report about the design and biological characterization of novel cell-penetrating peptides (CPPs) with selective suborganelle-targeting properties. The nuclear localization sequence N50, as well as the nucleoli-targeting sequence NrTP, respectively, were fused to a shortened version of the cell-penetrating peptide sC18. We examined cellular uptake, subcellular fate and cytotoxicity of these novel peptides, N50-sC18* and NrTP-sC18*, and found that they are nontoxic up to a concentration of 50 or 100 µM depending on the cell lines used. Moreover, detailed cellular uptake studies revealed that both peptides enter cells via energy-independent uptake, although endocytotic processes cannot completely excluded. However, initial drug delivery studies demonstrated the high versatility of these new peptides as efficient transport vectors targeting specifically nuclei and nucleoli. In future, they could be further explored as parts of newly created peptide–drug conjugates.

## Introduction

Various drugs act on targets that are located within the nucleus, the control center of the eukaryotic cell. A lipid bilayer membrane, which is perforated with nuclear pore complex structures through which the transfer of molecules is regulated, separates the nucleus from the cytosol. Macromolecules, like proteins, gain access to the nucleus by recognition of their nuclear localization sequences (NLS) by NLS-receptors, and following energy-dependent uptake processes. Several such natural occurring protein-derived NLS have been already identified and described [1]. Moreover, peptides that specifically target to subnuclear sites, e.g., nucleoli, have been characterized [2–3]. The nucleolus is formed at discrete chromosomal loci and its major role is the generation of ribosomal key components and assembly of the ribosomes [4]. Selective inhibition of the ribosomal machinery has been shown to be an effective anticancer therapeutic strategy [5]. That is why selective drug transport to the nucleoli has emerged a potent new strategy in anticancer drug development [6–7].

Based on these homing domains, a substantial number of sequences have been designed for addressing and delivering anticancer drugs to the nuclei and its subnuclear regions. Although several drugs might be delivered successfully inside a cell, they often fail since they are not able to reach their subcellular target. In order to circumvent adverse side effects, there is a need to develop suitable delivery vectors for the safe transport of drugs to the nucleus. Such nuclear-targeting sequences have already proven to be successful delivery tools. According to their often basic nature, they are also able to traverse the cellular membrane [8]. Based on this, these peptides have been added to the growing family of cell-penetrating peptides (CPPs). CPPs are able to overcome the cellular membrane and to enhance the intracellular uptake of CPP-modified molecules [9]. Usually, these peptides are relatively short (**≤**30 amino acids (aa)) and display an amphipathic or basic character. During the last 25–30 years, many different CPPs have been described and used for manifold applications like the delivery of nucleic acids, proteins, peptides, nanoparticles, small organic drugs, and others [10]. CPP conjugates can be generated by covalent conjugation between cargo and CPP or by forming non-covalent complexes. Notably, the mechanism of cell entry is still not fully understood, and can only hardly, if in any case, be predicted [9]. In fact, whereas one of the main mechanisms is endocytosis, there exist also CPPs that translocate through cellular membranes by direct penetration. The latter is described for those cases, where only small cargos are attached to the CPP [11]. We have designed a cell-penetrating peptide sequence, namely sC18, which we efficiently used in previous studies as drug transporter [12–17]. sC18 is composed of the last 16 *C*-terminal aa of the cationic antimicrobial peptide CAP18 [18]. When it comes in contact with lipid membranes, it forms a helical structure, probably supporting membrane interaction [19]. However, the main uptake mechanism that was observed followed endocytotic processes, although we have seen that sC18 is also able to enter cells directly to some extent, which is among others depending on the cell lines used [20].

For a further exploration and development of peptide–drug conjugates, peptide sequences that specifically accumulate at intracellular target sites are needed. CPPs have been already described as beneficial tools in the creation of anticancer drugs [21]. Within this study, we aimed to design novel efficient cell-penetrating peptides that preferentially locate within cell nuclei and subnuclear regions. For this, we generated peptide chimera consisting of a shortened version of the recently described sC18 peptide and a nuclear- or nucleolar-targeting sequence. These novel peptides proved to be very efficiently taken up by cancer cells and to accumulate within their target destinations. Beside a careful characterization concerning their uptake behavior, we used these peptides in an initial study for the delivery of the anticancer drug doxorubicin.

## Results and Discussion

### Peptide synthesis and analysis of the secondary structure

We chose two different nuclear-targeting sequences, on the one hand the N50 peptide, which was derived from the NF-κB/p50 subunit. N50 binds the adaptor protein importin-α at the nuclear envelope and triggers the uptake of the transcription factor NF-κB [22–23]. As second sequence we chose the NrTP sequence, which is a designed peptide coming from the rattlesnake toxin, called crotamine [3]. For both peptides, preferential accumulation within the nuclei has been already described. Moreover, for NrTP a subnuclear localization within the nucleoli has been reported. We designed peptide chimera by attaching these nuclear targeting sequences at the *N*-terminus of a shortened version of the sC18 peptide, namely sC18*, lacking the four *C*-terminal amino acids of sC18. Recently, we could show that sC18* was still able to enter cells, although with lower efficiency than sC18 itself [19–20]. However, to keep the final peptide sequence as short as possible, we used this minimalistic version. As control peptides, we additionally prepared the nuclear targeting sequences, as well as sC18* alone. All peptides were readily synthesized via Fmoc/*t-*Bu solid-phase peptide synthesis, purified, and analyzed by LC–MS methods as previously described [19–20]. Moreover, 5(6)-carboxyfluorescein (CF)-labeled versions were generated (Table 1).

**Table 1:** Names, sequences, molecular weights and net charges of the peptides that were investigated in this study. All peptides were obtained in >99% purity.

Name	Sequence^a^	MW_calcd_ [Da]	MW_exp_ [Da]	Net charge

sC18*	GLRKRLRKFRNK	1570.96	1571.36	+8
N50	VQRKRQKLMP	1282.61	1282.76	+5
N50-sC18*	VQRKRQKLMPGLRKRLRKFRNK	2836.51	2837.20	+12
NrTP	YKQCHKKGGKKGSG	1504.76	1505.03	+6
NrTP-sC18*	YKQCHKKGGKKGSGGLRKRLRKFRNK	3058.67	3059.31	+13

^a^All peptides are *C*-terminally amidated. For internalization studies, also 5(6)-carboxyfluorescein-labeled peptides were synthesized.

As shown in Table 1 and Figures S1–S4 (Supporting Information File 1), all peptides could be successfully synthesized in high purities.

First, we performed a structural analysis by diluting all peptides to a concentration of 20 μM in phosphate buffer (pH 7.0), with or without the presence of the secondary structure inducing solvent trifluoroethanol (TFE) [24].

**Figure 1:**
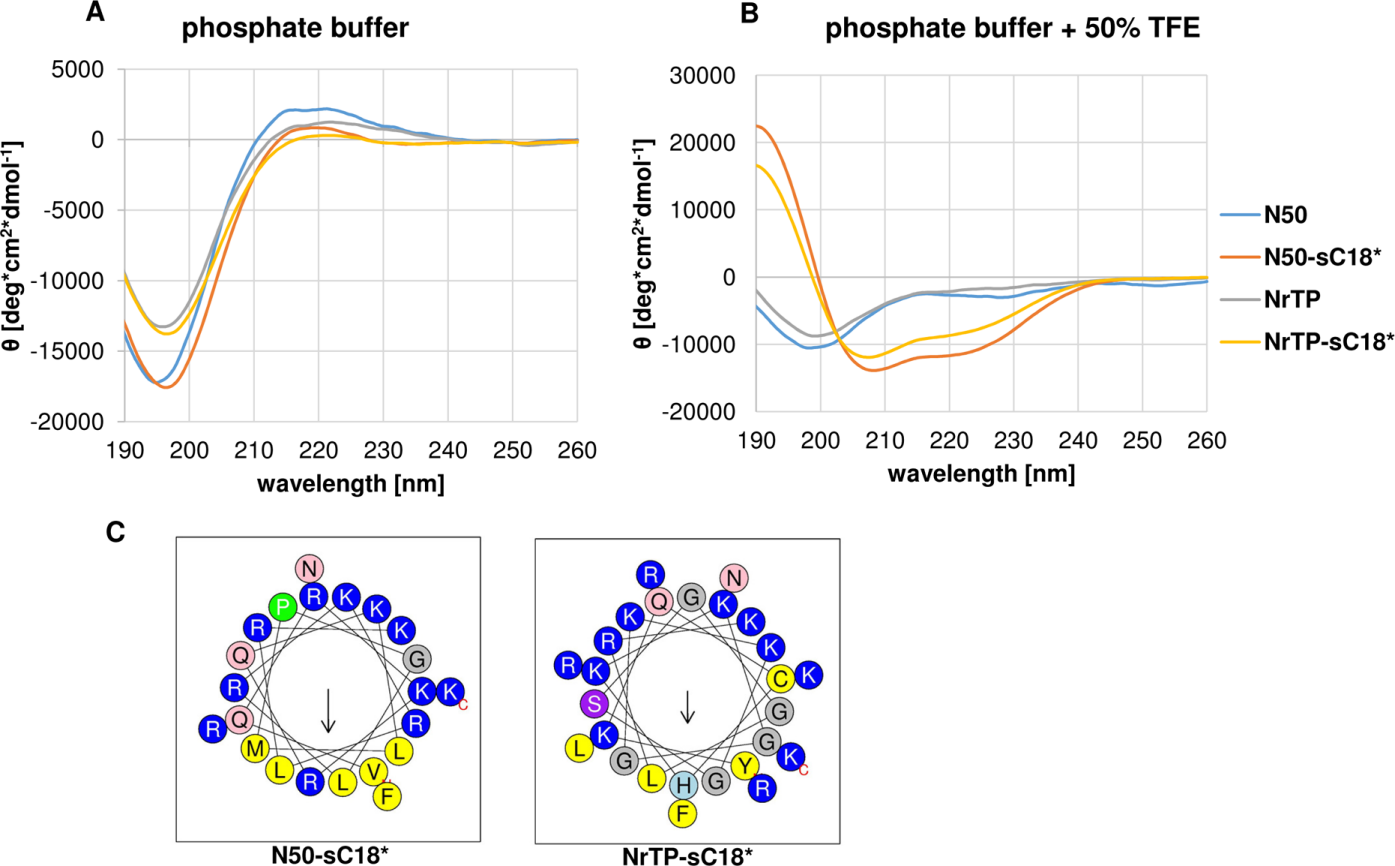
Circular dichroism spectra of the novel peptides solved in 10 mM phosphate buffer (pH 7) (A), or phosphate buffer with the addition of TFE (50%) (B). Peptide concentration was 20 µM. (C) Helical wheel projections of the peptides N50-sC18* and NrTP-sC18*, respectively [26].

As can be depicted from Figure 1, all peptides exhibited a random coil structure in phosphate buffer without TFE. In the presence of TFE, the peptides N50 and NrTP also exhibited a random coil structure, whereas N50-sC18* and NrTP-sC18* formed α-helices. This was also confirmed by the calculated R-values, which were 0.83 for N50-sC18* and 0.70 for NrTP-sC18* [25]. In agreement with our recent studies sC18* exhibited an α-helical character in TFE solution (data not shown) [19]. Thus, the helical character of the novel fusion peptides likely results from the sC18* part. Furthermore, N50-sC18* and NrTP-sC18* formed α-helices that showed amphipathic character with a clear hydrophilic and hydrophobic face (Figure 1C). This property might support the interaction with the plasma membrane.

### Cytotoxic profile of novel CPPs

**Figure 2:**
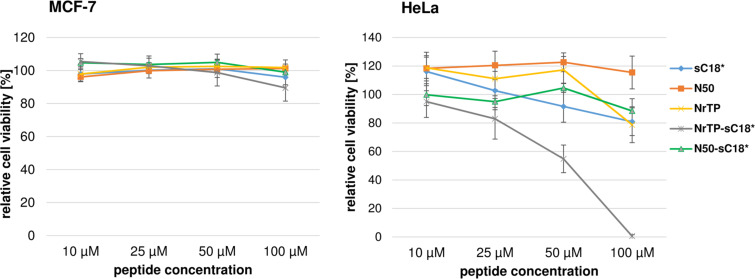
Cytotoxicity profiles of the peptides in MCF-7 and HeLa cells. Cells were incubated for 24 h with different concentrations of peptide solutions. Untreated cells served as negative control, cells treated with 70% ethanol as positive control. Values from the positive control were subtracted from all data, and the untreated cells were set to 100%; assays were performed with *n* = 3 in triplicate.

In the next step, the cytotoxicity profiles of the novel peptide chimera were investigated. Therefore, we chose two different cancer cell lines, namely breast cancer MCF-7 and cervix carcinoma HeLa cells, which were exposed for 24 h to various concentrations of the peptides sC18*, N50, N50-sC18*, NrTP and NrTP-sC18* (Figure 2).

We observed no toxic effects of the peptides when incubated with MCF-7 cells up to a concentration of 100 μM. Also after treating HeLa cells with the peptides up to a concentration of 50 μM, no significant toxicity could be observed for sC18*, N50, N50-sC18* and NrTP. Besides N50, all other peptide sequences did lower the amount of viable cells to an amount of around 80% at higher concentrations. For sC18* the results are in very good agreement to our former studies, in which we also examined the toxicity in other cell lines, like human epithelial kidney cells (HEK-293) and human colorectal adenocarcinoma cells (HCT-15) [19–20]. Notably, NrTP-sC18* seemed to affect cell viability at a concentration of 50 µM, and at higher concentrations, all cells were dead. To get a more detailed picture, we additionally determined the IC_50_ value of this peptide, NrTP-sC18*, when in presence of HeLa cells. An IC_50_ value of about 53.72 ± 4.79 µM was calculated after incubating the cells with various concentrations from 1 to 100 µM (Figure S5, Supporting Information File 1), demonstrating its high toxic effects in this cell line. Probably NrTP-sC18* interacts with distinct intracellular targets, but this has to be elucidated in further studies. However, all following uptake experiments were conducted at peptide concentrations between 1 and 10 μM, where no significant effect on cell viability was observed in both cell lines.

### Cellular uptake studies

**Figure 3:**
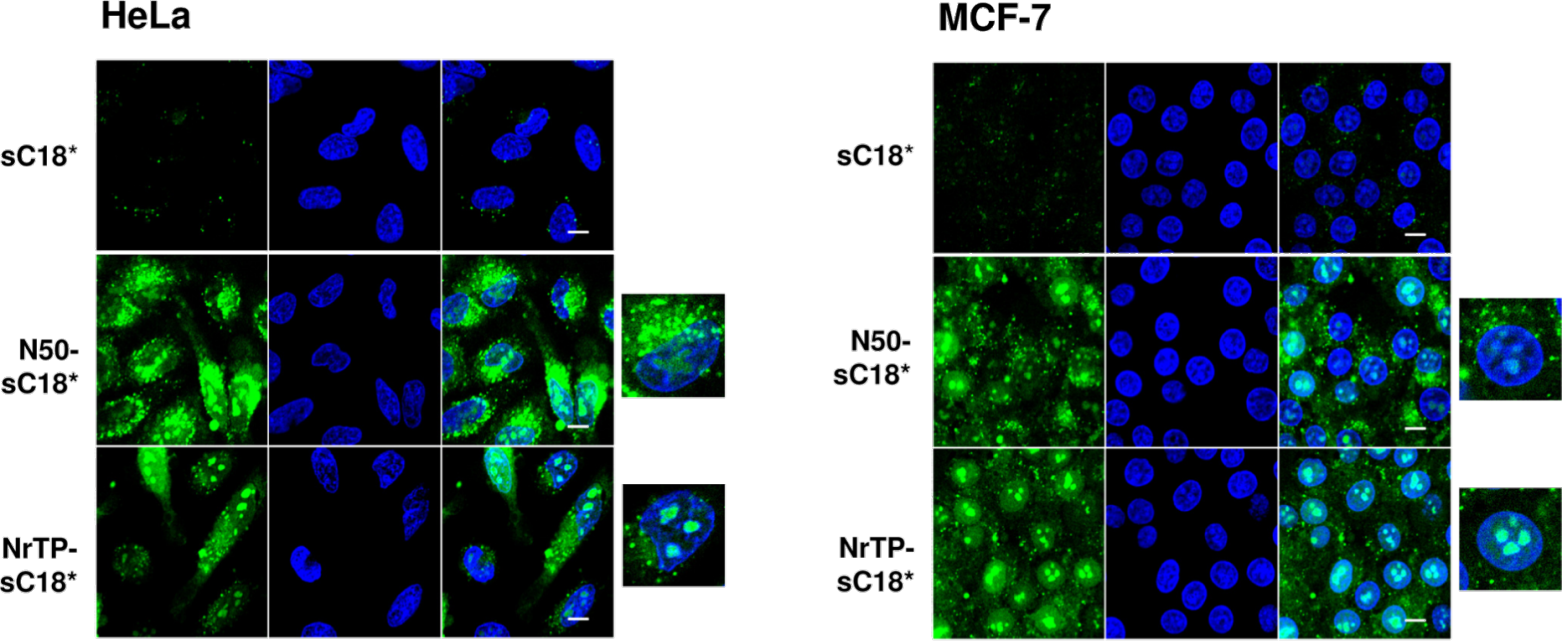
Cellular uptake in HeLa and MCF-7 cells. Cells were incubated for 30 min with 10 µM of CF-labeled peptide solutions. Green: CF-labeled peptide; blue: Hoechst 33342 nuclear stain; scale bar is 10 µm.

Next, we analyzed the intracellular fate of the new peptide variants using confocal fluorescence microscopy. Thus, MCF-7 and HeLa cells were incubated with 10 µM peptide solutions at 37 °C and inspected after 30 min (Figure 3).

Surprisingly, both new peptide variants, N50-sC18* and NrTP-sC18*, entered the cells extremely efficiently compared to sC18* alone. For sC18*, only small dots were detectable, which were probably representing vesicles, since an endocytotic uptake pathway for this CPP and its longer version sC18 was already demonstrated [13,18–19]. In addition, the nuclear localization sequence N50 alone was not noticeable present within both cell lines at the tested concentration (Figure S6, Supporting Information File 1), not even after a longer incubation period of two hours (data not shown). For NrTP alone, a slight fluorescent signal was visible in the nucleoli of MCF-7 cells (Figure S6, Supporting Information File 1). Notably, N50-sC18* was distributed within the whole cell cytosol, and accumulated particularly around the nucleus. In addition to that, a large fraction was also centered within the nuclei and nucleoli. For the fusion peptide NrTP-sC18* a strong accumulation within the nucleoli of both cell lines was visible. Thus, former results about the preferential localization within the nucleolar region of the peptide NrTP alone could be confirmed also for NrTP-sC18* [3].

**Figure 4:**
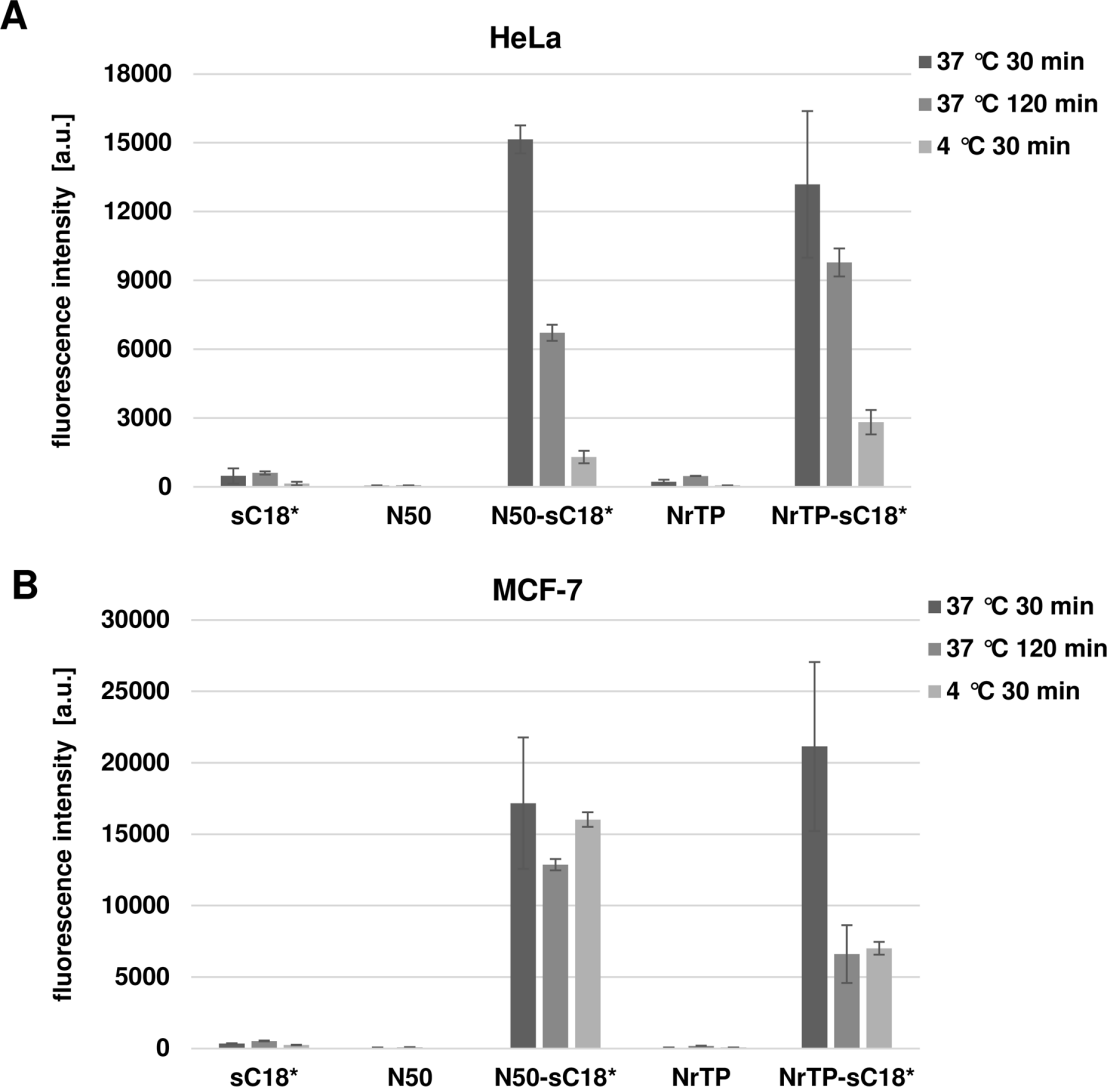
Cellular uptake in MCF-7 and HeLa cells was quantified by flow cytometry. Cells were incubated with 10 µM peptide solutions for 30 min at 37 °C.

We then quantified the cellular uptake by using flow cytometry. As expected, the novel peptides N50-sC18* and NrTP-sC18* were characterized by an extremely high uptake compared to the CPP sC18*, as well as the nuclei targeting sequences alone (Figure 4).

Rádis-Baptista et al. recently reported about the effective uptake of rhodamine B-labeled NrTP in different tumor cell lines [3,27]. Within their studies, the authors used higher concentrations, longer incubation times and other cell lines, what could probably explain the different results obtained in our study. In fact, it is very likely that working with increased concentrations of the NrTP sequence could probably improve the cell-penetrating capability of this peptide. Also for the N50 sequence alone, the internalization ability in several different cell lines was already determined [28]. In this case, the uptake turned out to be very low, what is in agreement with our results. The observed enhanced cellular uptake of the novel chimeric peptides might be due to an increased amount of positive charges caused by the presence of more lysine and arginine residues within the sequences. These effects were already described by other groups working with highly cationic CPPs [29–32]. Moreover, the formation of amphipathic α-helices is often one major factor for efficient peptide/lipid interaction, initiating the following internalization process [33]. We have observed that CPP attachment to the nuclei-targeting sequences promotes the formation of such favored secondary structures (e.g., α-helices). Hence, this could be one important key factor for the detected efficient cellular uptake.

**Figure 5:**
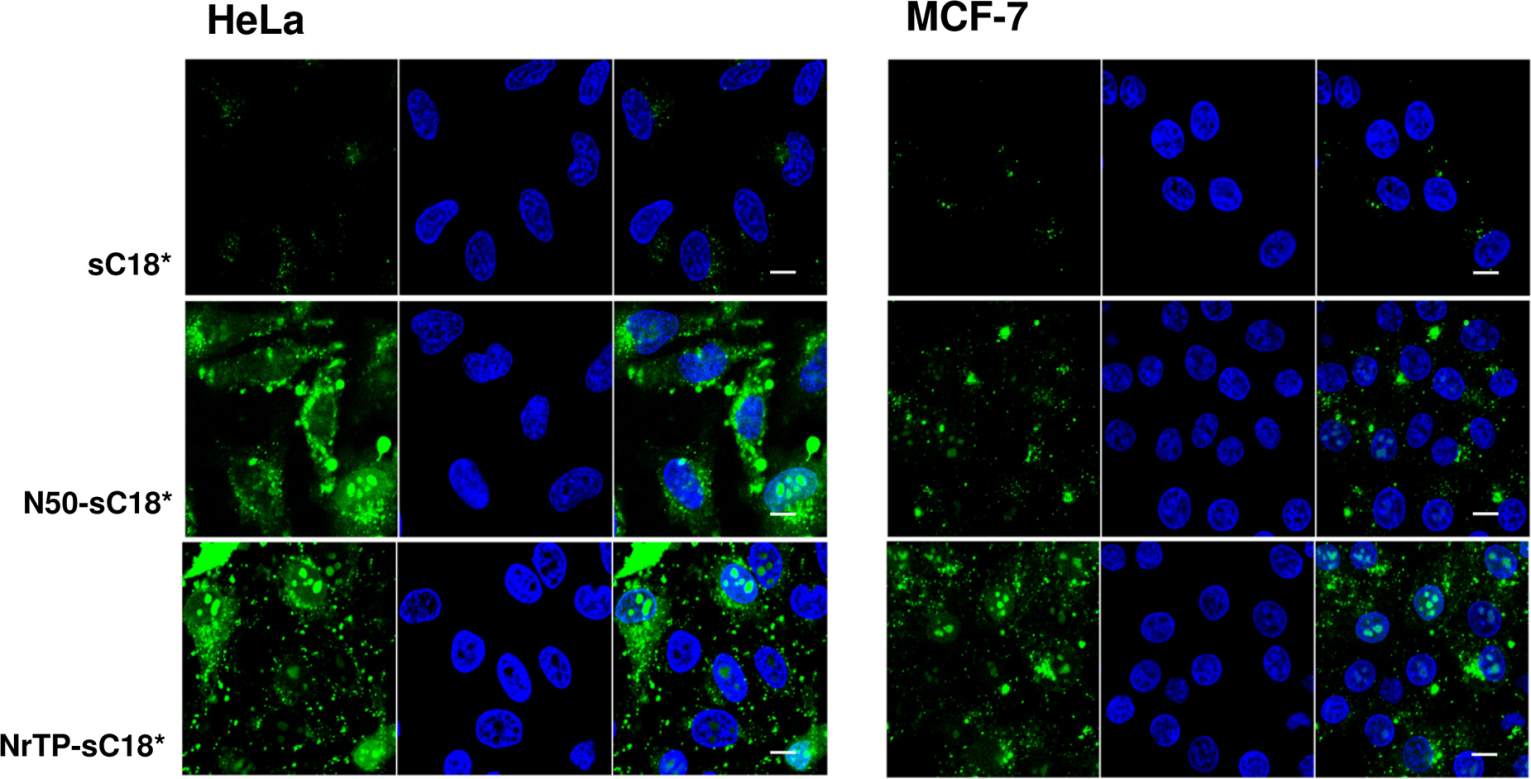
Distribution pattern of the peptides in HeLa and MCF-7 cells when incubating 10 µM CF-labeled peptide solutions for 120 min at 37 °C. Green: CF-labeled peptide; blue: Hoechst 33342 nuclear stain; scale bar is 10 µm.

Furthermore, we observed that still after 2 hours of incubation with the peptides, strong green signals were visible (Figure 5). In contrast to the pictures taken after 30 min, it seemed that the peptides also formed aggregated structures within the cytosol, beside the fraction that is still localizing in the nuclei. Qian et al. recently discussed such structures as a result of peptide/lipid aggregation [34]. However, since only the fluorescence of the fluorophore can be detected, it can of course not be ruled out that degradation of the peptides has been already started. Quantifying the amount of the novel peptides after 120 min demonstrated further that the uptake was lower compared to 30 min, but still very high compared to sC18* alone (Figure 4).

Anyway, as the internalization with 10 μM of the peptides was quite high and the accumulation, especially for N50-sC18* was not precisely detectable in HeLa cells caused by an intense green signal in the whole cell, we performed experiments using a lower peptide concentration of 1 μM. Next to this, also the shorter peptides, namely sC18*, N50 and NrTP alone were tested at these concentrations for 30 and 120 min. Hereby, no uptake at all was detected (data not shown). In contrast, both fusion peptides were able to efficiently internalize into HeLa cells (Figure S7, Supporting Information File 1). Obviously, the uptake was less compared to that one at a concentration of 10 μM. N50-sC18* was diffusely distributed within the cytosol and the nuclei of HeLa cells, and after 120 min it mainly accumulated within the nuclei. In contrast to this, the uptake of 1 μM of NrTP-sC18* indicated that the peptide accumulates in endosomes after cellular uptake. Interestingly, at this concentration, the peptide was neither detectable in the nuclei nor in the nucleoli. Probably the concentration of NrTP-sC18* was not high enough to escape from the vesicles and to reach the nuclei. This might indicate that a certain concentration threshold is indispensable for efficient internalization, a phenomenon that was already discussed for other CPPs [29,35].

Considering all these observations, it was presumed that the chimeric peptides enter the cells concentration-dependent by direct penetration or by endocytosis, followed by an endosomal release, which could already be shown for other sC18 derived CPP variants [20].

**Figure 6:**
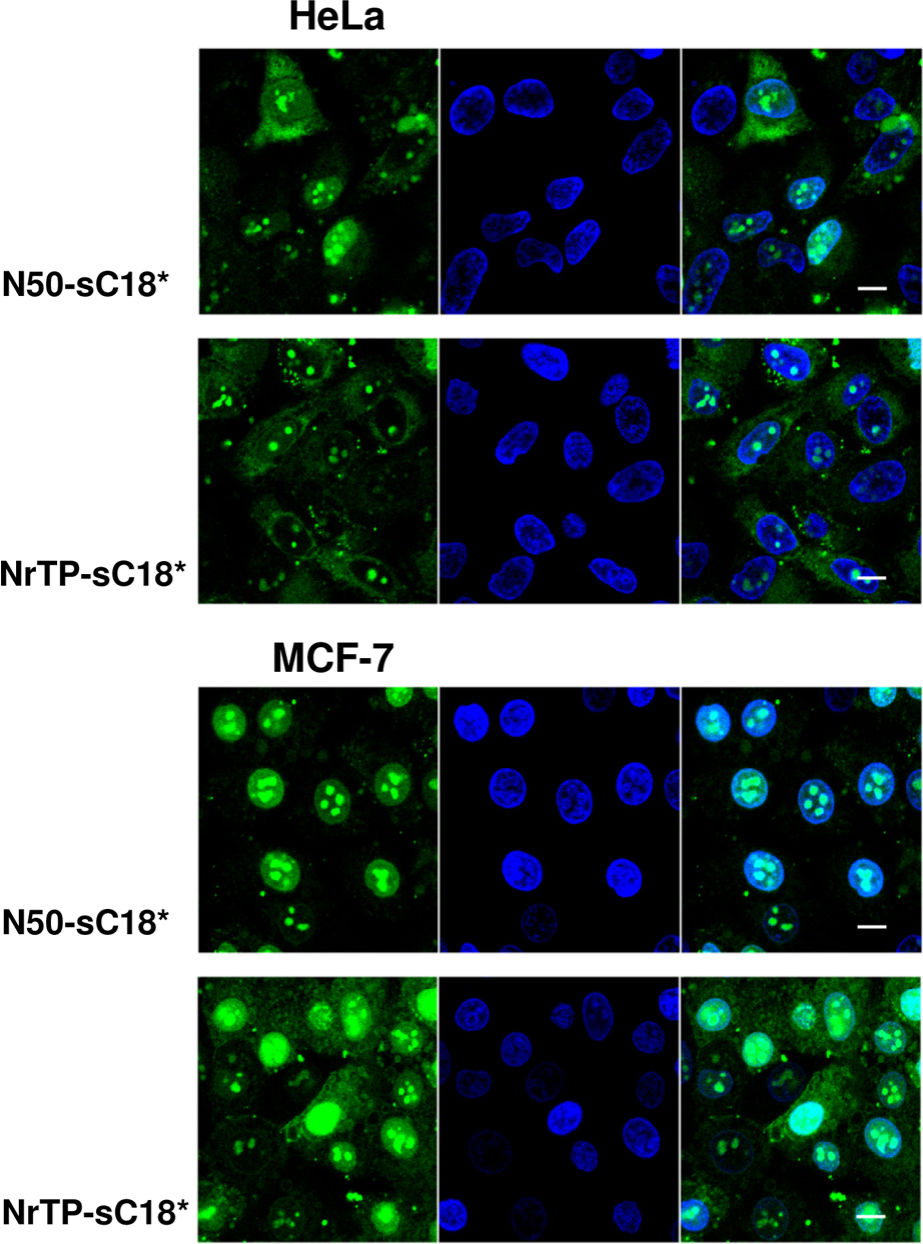
Cellular uptake in HeLa and MCF-7 cells when incubating the cells at 4 °C for 30 min with the CF-labeled chimeric peptides (10 µM). Green: CF-labeled peptide; blue: Hoechst 33342 nuclear stain; scale bar is 10 µm.

To get an idea about the involvement of endocytotic processes during peptide internalization, uptake studies at 4 °C were performed (Figure 4 and Figure 6). Hereby, energy-dependent pathways are usually suppressed and direct peptide translocation can be observed [36]. After treating MCF-7 and HeLa cells for 30 min with the peptides at 4 °C, both fusion peptides were distributed within the cytoplasm and also accumulated in the cell nuclei and nucleoli. This observation supported the idea of cellular entry via direct penetration when using a concentration of 10 µM. In MCF-7 cells, NrTP-sC18* was also evenly distributed throughout the whole cell, including strong accumulation in the nuclei. Notably, N50-sC18* was mainly detectable within the nuclei. Thus, the fusion peptides might indeed enter the cells via direct translocation, although also energy-dependent uptake pathways cannot be ruled out, especially when lower concentrations are applied. However, for both peptides N50-sC18* and NrTP-sC18*, we could prove that they potently address the nuclei/nucleoli. Moreover, as can be depicted from Figure 4, the peptides were taken up to a significant less extent in both cell lines, when cells were incubated at 4 °C. This points again to an involvement of energy-dependent uptake pathways. Notably, the uptake was not completely reduced, indicating the involvement of direct entry processes that may play a role during cellular uptake.

### Use of novel peptides as cargo delivery systems

In the last experiment, we investigated if the peptides could be used to enhance the efficacy of an anticancer drug. Therefore, HeLa and MCF-7 cells were exposed to the chemotherapeutic drug doxorubicin (DOX) that is already clinically applied in cancer therapy [37]. Doxorubicin interacts with DNA by intercalation and thereby inhibits the macromolecular biosynthesis [38]. Instead of covalent conjugation of the drug, we aimed to investigate the effect of the fusion peptides on drug delivery and efficacy within co-administration. Indeed, the covalent binding of doxorubicin to different CPPs was already reported and the induction of cell death in various cell lines has been observed [39–40]. However, the non-covalent approach of co-administration is often favored owing to the ease of preparation and a higher capacity of drug that can be administered. Such a combination therapy of DOX and a tumor-penetrating peptide has been recently investigated in vivo using clinically relevant tumor models [41].

**Figure 7:**
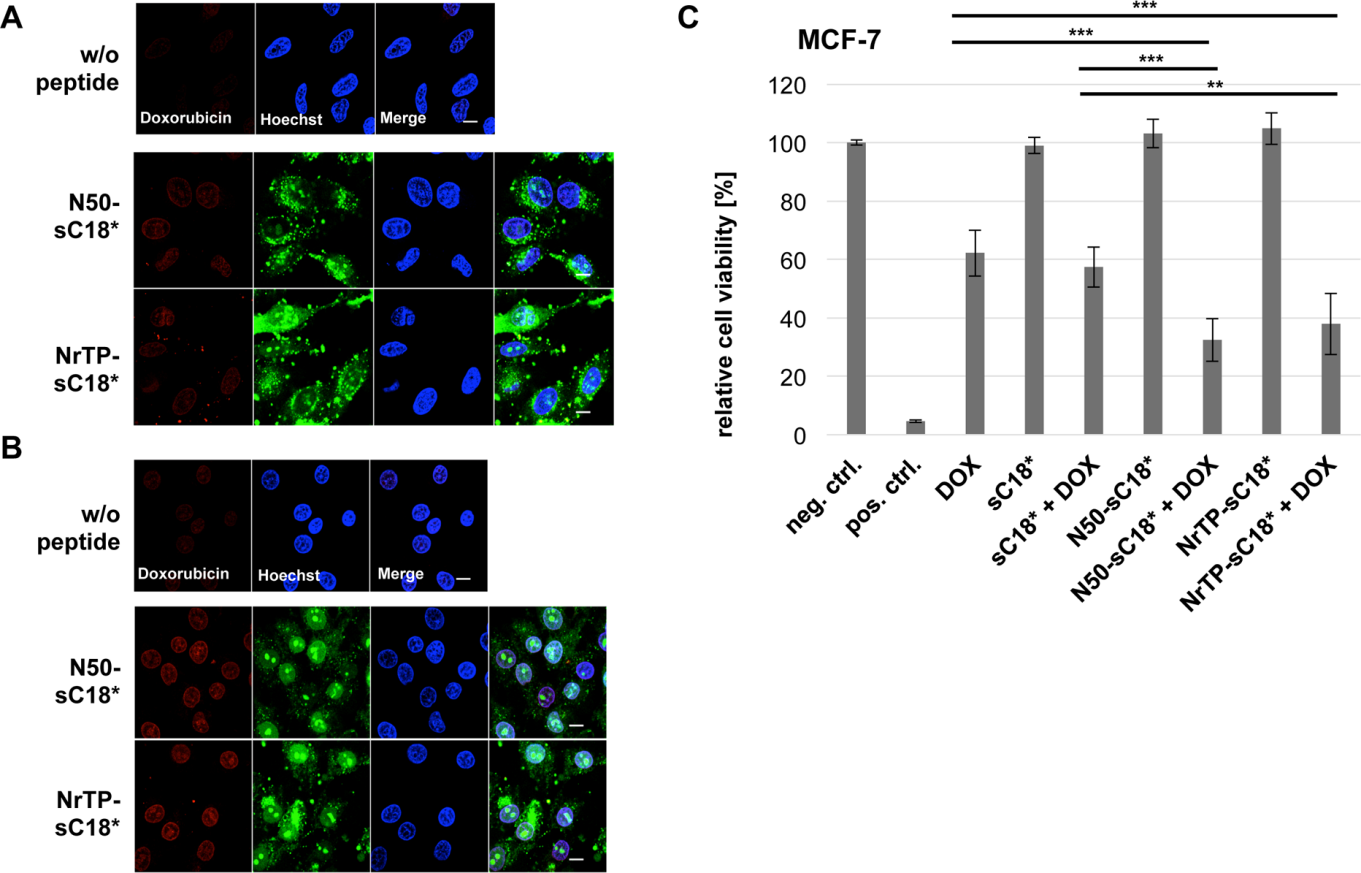
Uptake and delivery of DOX into HeLa and MCF-7 cells. Fluorescence microscopic images after 30 min incubation in HeLa (A) and MCF-7 (B) cells with 10 μM CF-labeled peptides N50-sC18* or NrTP-sC18* co-incubated with 10 μg/mL doxorubicin at 37 °C, respectively. Cells treated with DOX alone served as negative control; Green: CF-labeled peptide; blue: Hoechst 33342 nuclear stain; red: doxorubicin; scale bar is 10 µm. (C) Cells were incubated for 48 h with DOX (1 µg/mL), or solutions out of peptides (10 µM) and DOX (1 µg/mL), or peptide solutions (10 µM) alone. Untreated cells served as negative control, cells treated for 10 min with 70% ethanol as positive control. Experiments were conducted in triplicate with *n = 2*.

Doxorubicin is known to be fluorescent [42] and this property was used to test if the peptides were able to enhance the intracellular uptake of the drug. Therefore, solutions out of DOX and the sequences N50-sC18* and NrTP-sC18* were incubated with HeLa and MCF-7 cells, respectively, and observed for red fluorescence afterwards (Figure 7A and 7B).

Minor red fluorescence could be detected in the negative control (DOX only), indicating that the chemotherapeutic drug was also able to translocate in the cells by itself at the used concentration of 10 µg/mL. Apart from that, it is visible that DOX fluorescence is increased when co-administered with the novel peptides (Figure 7A and 7B). Since this effect was more intense in MCF-7 cells, these cells were used for a following cytotoxicity assay. Herein, the drug alone (1 μg/mL) or in presence of 10 μM solutions of the peptides sC18*, N50-sC18* and NrTP-sC18*, respectively, were incubated for 48 h with MCF-7 cells. As can be depicted from Figure 7C, treatment with doxorubicin alone reduced the amount of viable cells to about 60%. The peptides alone were not toxic at a concentration of 10 μM, which was already demonstrated (Figure 2). In contrast, when co-incubating N50-sC18* and NrTP-sC18* with DOX, the toxic effect of the drug could be significantly improved. After co-treatment with NrTP-sC18*, the number of viable cells was decreased to 40% and for N50-sC18* to 30%, although no significant difference in activity of both peptides could be determined. Notably, the peptides NrTP-sC18* and N50-sC18* are more efficient than the sC18* sequence alone. While the presence of sC18* could not enhance the efficacy of DOX, the combination of sC18* with the nuclear targeting sequences N50 and NrTP led to an improved drug uptake, and very likely to an increased accumulation of the drug within the nuclei.

## Conclusion

In summary, we presented herein the design and activity of novel CPPs, namely N50-sC18* and NrTP-sC18*. Their low cytotoxicity in combination with their high internalization efficiency and target selectivity make these novel peptides promising new transport shuttles. Having shown the great potency of CPP in anticancer drug research, these peptides could be used in future for the development of further innovative and highly effective peptide–drug conjugates.

## Experimental

### Materials

All *N*_α_-Fmoc protected amino acids (aa) were purchased from IRIS Biotech (Marktredwitz, Germany). Other chemicals and consumables including 1-[bis(dimethylamino)methylene]-1*H*-1,2,3-triazolo[4,5-*b*]pyridinium 3-oxide hexafluorophosphate (HATU), *N*,*N*-diisopropylethylamine (DIPEA), acetonitrile (ACN), and trifluoroacetic acid (TFA), dimethylformamide (DMF), *N*-methylpyrrolidine (NMP), Oxyma, *N*,*N*’-diisopropylcarbodiimide (DIC), doxorubicin (DOX) and 5(6)-carboxyfluorescein (CF) were derived from Fluka (Taufkirchen, Germany), Merck (Darmstadt, Germany), Sarstedt (Nümbrecht, Germany), Sigma-Aldrich (Taufkirchen, Germany) and VWR (Darmstadt, Germany).

### Peptide synthesis

All peptides were synthesized using a combination of standard Fmoc/*t*-Bu solid-phase peptide synthesis (SPPS) on a Syro I peptide synthesizer (MultiSynTech, Bochum, Germany) and manual coupling protocols according to previous works [17,19–20]. Peptides were generated on a Rink amide resin (loading 0.48 mmol/g) yielding *C*-terminally amidated molecules.

All syntheses were performed in open polypropylene reactor vessels (2 mL syringes) stocked with a fritted filter disc. All aa were dissolved in DMF except from phenylalanine that was dissolved in NMP. Amino acids were coupled in 8-fold excess and every coupling step was performed twice using Oxyma/DIC as activating reagent. Every coupling step proceeded for 40 min. After complete synthesis, the samples were washed with CH_2_Cl_2_, MeOH and Et_2_O and the resin beads were dried in the Speedvac.

5(6)-Carboxyfluorescein (CF) was coupled with 3 equiv HATU and DIPEA in DMF for 2 h at rt as described previously [20]. CF-polymers were cleaved by treatment with 20% piperidine for 45 min. The successful coupling was verified by a Kaiser test [43].

To cleave the peptides from the resin, a mixture of triisopropylsilane (TIS), H_2_O and concentrated trifluoroacetic acid (TFA) (1:1:38 v/v/v) was added for 3 h. Afterwards, the peptides were precipitated in ice cold diethyl ether, washed and lyophilized from water/*tert-*butanol (3:1 v/v). Then, peptides were analyzed by RP-HPLC/ESI-MS on a Kinetex C18 column (100 × 4.6 mm; 2.6 μm/100 Å) using linear gradients of 10–60% B in A (A = 0.1% FA or TFA in water; B = 0.1% FA or TFA in acetonitrile) over 20 min and a flow rate of 0.6 mL·min^−1^. Further purification of the peptides was achieved by preparative HPLC on RP18 Phenomenex column (Jupiter Proteo, 250 × 15 mm, 4 μm/90 Å) using linear gradients of 10–60% B in A (A = 0.1% TFA in water; B = 0.1% TFA in acetonitrile) over 45 min and a flow rate of 6 mL·min^−1^. All peptides were obtained with purities >99%.

### CD spectroscopy

All peptides were analyzed in 10 mM potassium phosphate buffer (pH 7.0) with or without the addition of TFE (1:1 dilution) using a peptide concentration of 20 µM. Peptides were measured in a 0.1 cm quartz cuvette with a sensitivity of 100 mdeg in the range from 260 to 180 nm in 0.5 nm intervals. The scanning mode was continuous and a scanning speed of 50 nm/min was chosen. The results of pure buffer were subtracted from the spectra of the peptides. The ratio between the molar ellipticity at 222 nm and 207 nm was used to confirm an α-helical structure of peptides [25].

### Cell culturing

All cell lines were grown in sterile culture dishes in a CO_2_ incubator (5% CO_2_) at 37 °C. HeLa and MCF-7 were grown in RPMI-1640 medium containing 10% fetal bovine serum (FBS) and 4 mM L-Gln. HeLa and MCF-7 cells were not used above the 40th passage.

For seeding a defined number of cells, they were removed with trypsin/EDTA solution and a hemocytometer was used for cell counting.

### Cell viability assay

A total volume of 200 μL of cells (HeLa 40’000, MCF-7 50’000 cells per well) were seeded in 96-well plates and grown to 70–80% confluency. Afterwards, they were incubated with peptide or doxorubicin solutions (diluted in serum-free medium) in a total volume of 100 μL. In the wells that served as positive and negative controls, the medium was replaced by fresh medium without FBS. The plates were incubated for 24 h or 48 h at 37 °C. Afterwards, the positive control was treated with 100 μL of 70% EtOH for 10 min, and then all cells were washed with PBS. Cells were covered with 100 μL of a 10% resazurin solution in medium without FBS and incubated for 1–2 h at 37 °C. Afterwards, fluorescence was quantified by using a Tecan infinite M200 plate reader (excitation: 550 nm, emission: 595 nm).

To achieve comparably results, the positive control was subtracted from all data and the negative control was set to 100%, so that the results of the peptide-treated cells represent relative cell viability values in %. Experiments were done in triplicate.

### Microscopy

For microscopic analyses, a confocal laser scanning system (Nikon D-Eclipse C1) with an inverted microscope (Nikon Eclipse Ti) was used. Pictures were taken with a 60× oil immersion objective (N.A. 1.4, Plan APO VC; Nikon) using the software EZ-C1 3.91 from Nikon.

Cells were seeded in 350 μL medium in an 8-well ibidi plate (HeLa 45,000, MCF-7 70,000 cells per well) and were grown to 70–80% confluency. Then, the medium was removed and the cells were treated with the peptides and substances in various concentrations for the requested time. Cells were incubated at 4 °C or 37 °C and 10 min prior to the end of incubation 0.6 μL of Hoechst stain (bisbenzimide H33342, 1 mg/mL in H_2_O, sterile filtered) was added to each well to stain the cell nuclei. After removing the solutions, cells were quenched with 200 μL of 150 μM trypan blue solution (in acetate buffer) for 30 s. The stain was removed and the cells were washed twice with medium. After adding 300 μL of fresh medium, pictures were taken using a fluorescence confocal microscope. Images were edited in Image J 1.43m.

### Flow cytometry

Cells were seeded in 24-well plates (HeLa 170,000, MCF-7 200,000 cells per well) and grown to 70–80% confluency. Then, cells were treated with 400 μL of peptide solutions dissolved in serum-free medium for the appropriate time at 4 °C or 37 °C. Afterwards, the cells were washed twice with PBS and detached with Trypsin-EDTA 1× in PBS without phenol red for 3–5 min followed by adding 800 μL of indicator-free medium. Cells were resuspended and 200 μL of the suspension were transferred to a 96-well plate for measuring the fluorescence in the Guava^®^ easyCyte flow cytometer (Merck). In each sample, 10,000 cells were measured and each experiment was done in triplicate. Cells treated with medium only served as negative control and their fluorescent signal was subtracted from all other samples in each set of experiment.

## Supporting Information

File 1 Additional information.
