# Supplementary material for: Design and biological characterization of novel cell-penetrating peptides preferentially targeting cell nuclei and subnuclear regions
Source: Beilstein J Org Chem. 2018 Jun 7;14:1378–88. doi: 10.3762/bjoc.14.116 (PMC6009097; doi:10.3762/bjoc.14.116)
Supplement: File 1 — Additional information. [file Beilstein_J_Org_Chem-14-1378-s001.pdf]

Supporting Information

for

Design and biological characterization of novel cell-penetrating peptides preferentially targeting cell nuclei and subnuclear regions

Anja Gronewold[1], Mareike Horn[1] and Ines Neundorf*[1]

Address: [1]Department of Chemistry, Biochemistry, University of Cologne, Zuelpicher Str. 47a, 50674 Cologne, Germany

Email: Ines Neundorf - ines.neundorf@uni-koeln.de

* Corresponding author

Additional information

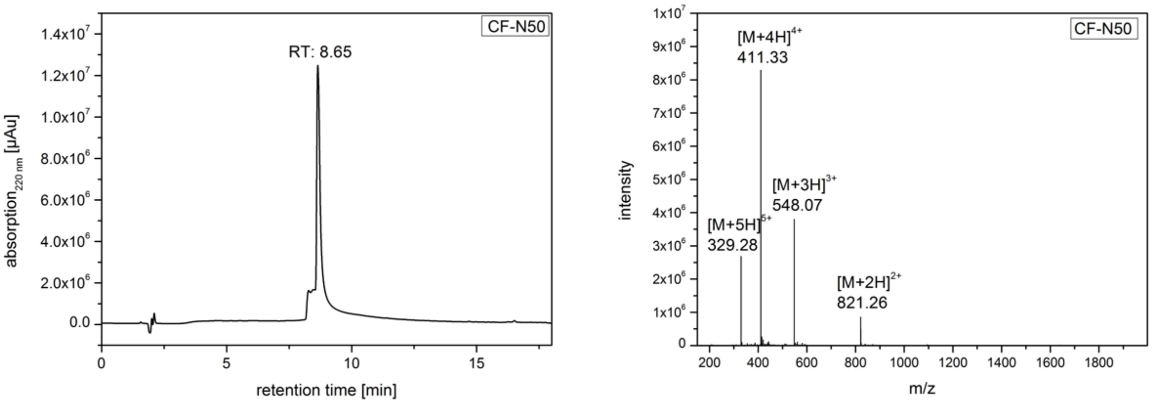

Figure S1: UV-chromatogram and ESI–MS spectrum of CF-labeled N50 after purification. The sample was recorded using a gradient from 10 to 60% ACN in water (incl. 0.1% TFA) within 15 min (for further details refer to experimental part). The shoulder at RT 8.5 in the UV-chromatogram corresponds to oxidized product.

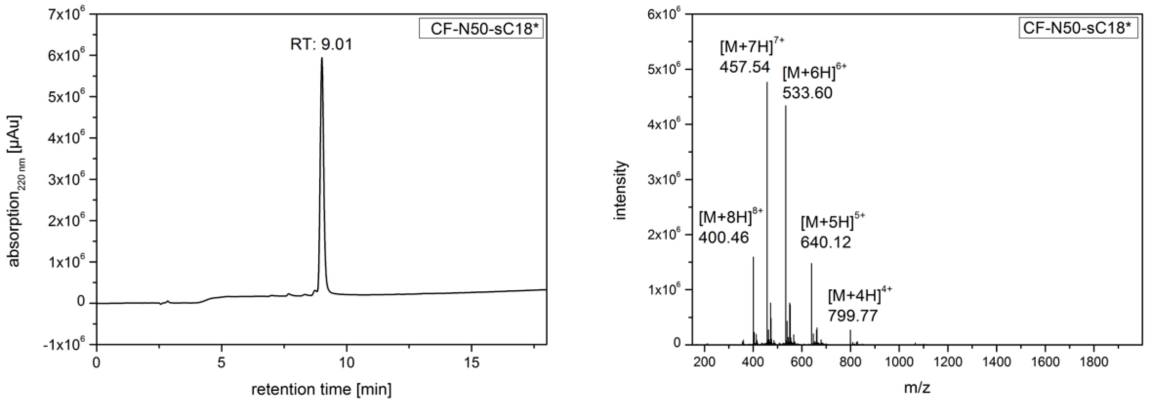

Figure S2: UV-chromatogram and ESI–MS spectrum of CF-labeled N50-sC18* after purification. The sample was recorded using a gradient from 10 to 60% ACN in water (incl. 0.1% TFA) within 15 min (for further details refer to experimental part).

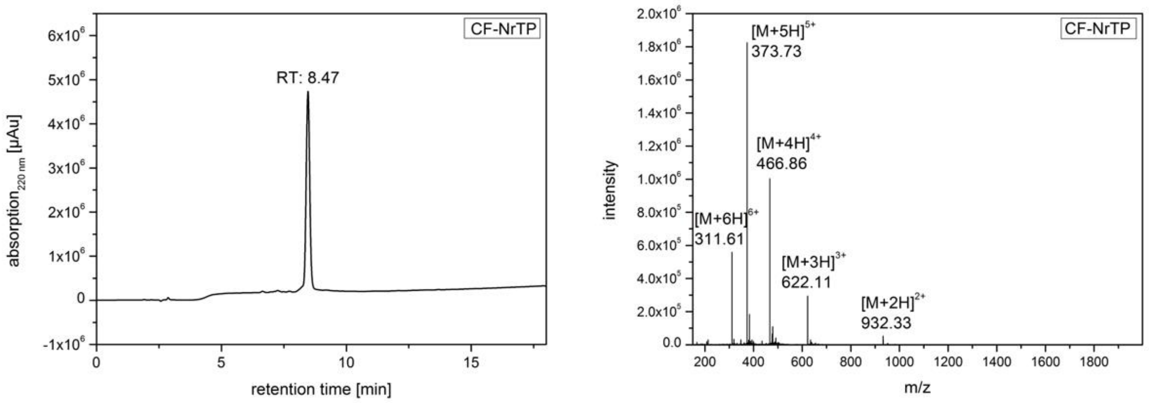

Figure S3: UV-chromatogram and ESI–MS spectrum of CF-labeled NrTP after purification. The sample was recorded using a gradient from 10 to 60% ACN in water (incl. 0.1% TFA) within 15 min (for further details refer to experimental part).

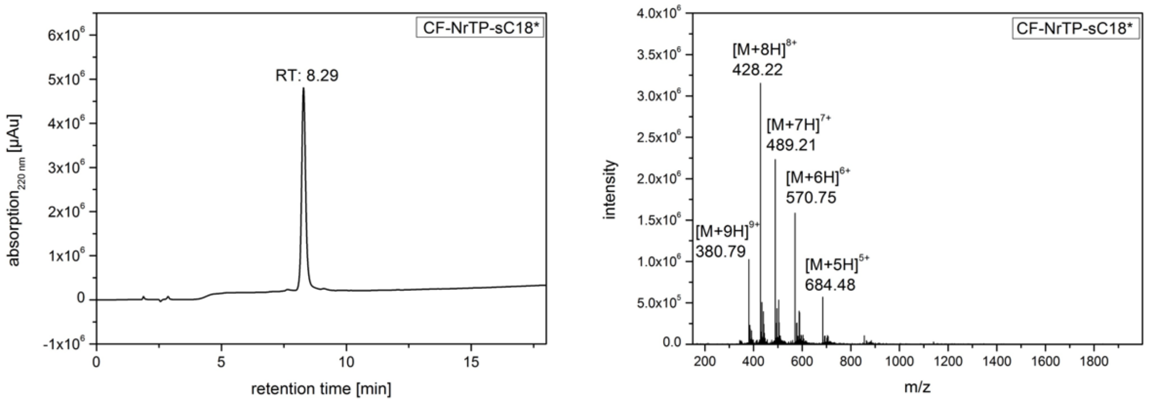

Figure S4*:* UV-chromatogram and ESI–MS spectrum of CF-labeled NrTP-sC18* after purification. The sample was recorded using a gradient from 10 to 60% ACN in water (incl. 0.1% TFA) within 15 min (for further details refer to experimental part).

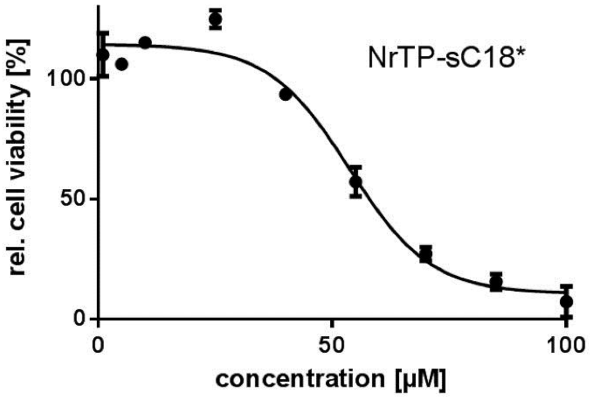

Figure S5*:* IC_{50} determination of NrTP-sC18* in HeLa cells (IC_{50} = 53.72 ± 4.79 µM). Peptide solutions of 1, 5, 10, 25, 40, 55, 70, 85 and 100 µM were incubated with HeLa cells (24 h, 37 °C). Afterwards a resazurin-based cell viability assay was performed according to the experimental part.

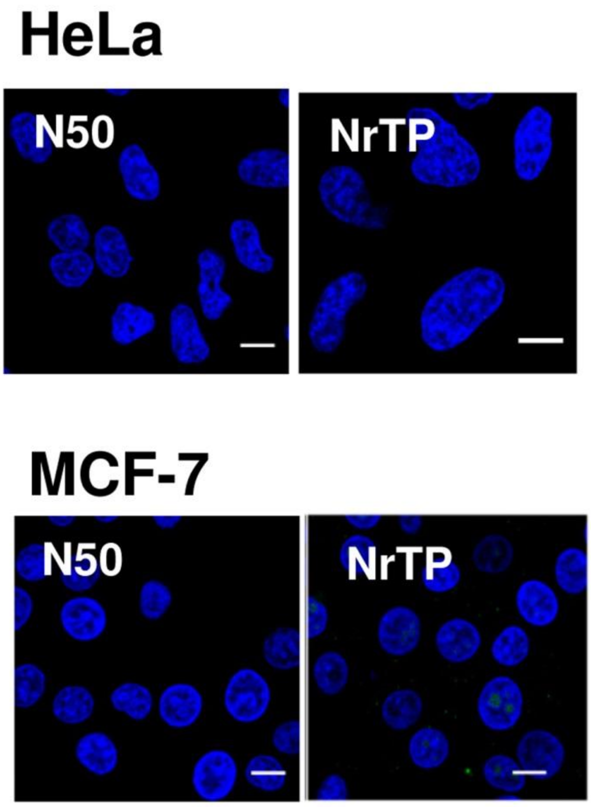

Figure S6: Cellular uptake of the control peptides N50 and NrTP in HeLa and MCF-7 cells. Cells were incubated for 30 min with 10 µM of CF-labeled peptide solutions. Blue: Hoechst 33342 nuclear stain; scale bar is 10 µm.

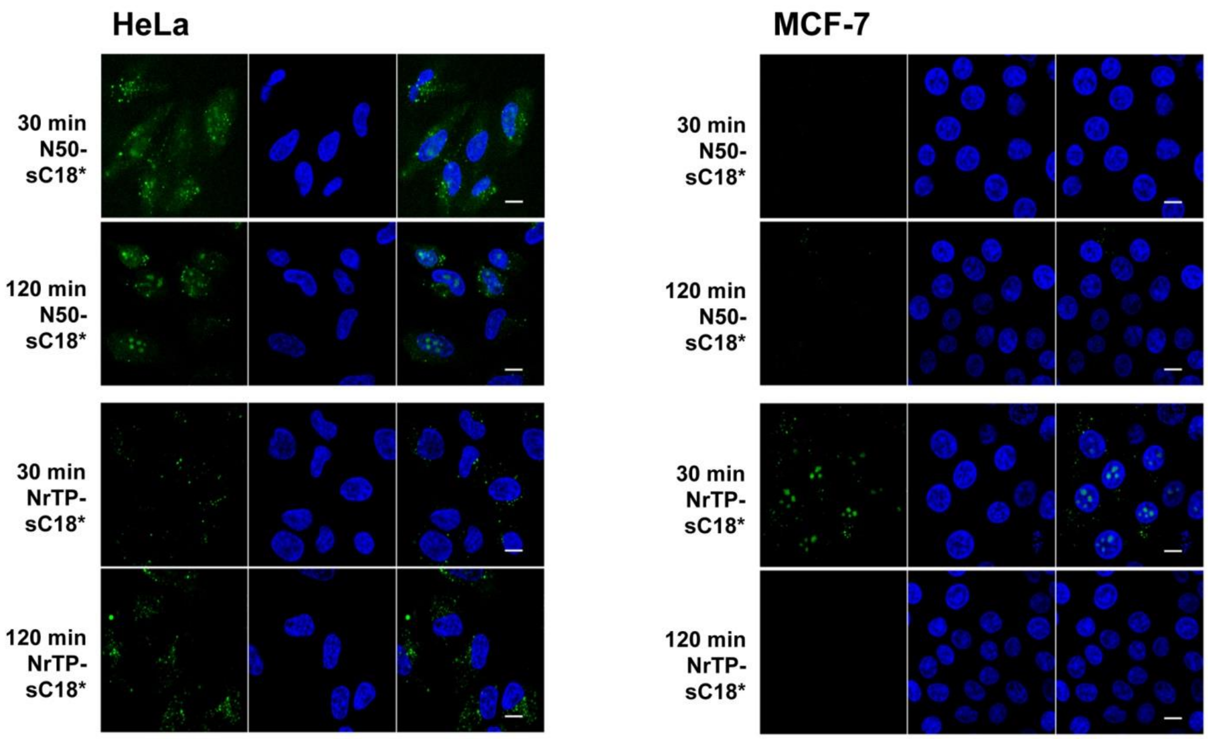

Figure S7: Internalization of the peptides in HeLa and MCF-7 cells. Cells were treated with 1 µM CF-labeled peptide solutions for 30 or 120 min, respectively, at 37 °C. Green: CF-labeled peptide; blue: Hoechst 33342 nuclear stain; scale bar is 10 µm.
